# Molecular Insights into the Synergistic Effect of Nano-Hydroxyapatite and *L-PRF* on Osteoporotic Osseointegration: An In Vivo Gene Expression Study

**DOI:** 10.3390/jfb17050250

**Published:** 2026-05-17

**Authors:** Ana Carolina Loyola Barbosa, José Augusto Gabarra Júnior, Lilian Eslaine Costa Mendes da Silva, Fernando Nóbrega, Edmara Tatiely Pedroso Bergamo, Bruna Ghiraldini, Roberto Sales e Pessoa, Michel Reis Messora, Sergio Scombatti de Souza

**Affiliations:** 1 School of Dentistry of Ribeirão Preto, University of São Paulo, Ribeirão Preto 14040-904, SP, Brazil; ana.barbosa@sinimplantsystem.com (A.C.L.B.); josegabarrajr@gmail.com (J.A.G.J.); nobrega.fernando@hotmail.com (F.N.); m.messora@forp.usp.br (M.R.M.); 2 Research & Development Department, S.I.N. Implant System, São Paulo 03033-021, SP, Brazil; bruna.ghiraldini@sinimplantsystem.com (B.G.); rp@inpes.com.br (R.S.e.P.); 3 Ribeirão Preto Medical School, University of São Paulo, Ribeirão Preto 14049-900, SP, Brazil; lilianeslaine@fmrp.usp.br; 4 Department of Biomaterials and Biomimetics, New York University, New York, NY 10010, USA; edmaratatiely@gmail.com; 5 Department of Periodontology and Implantology, School of Dentistry, University Centre of Triangulo-UNITRI, Uberlândia 38411-106, MG, Brazil

**Keywords:** nano-hydroxyapatite, L-PRF, osseointegration, osteoporosis, molecular rescue, gene expression

## Abstract

Poor bone quality in osteoporotic patients remains a major challenge for achieving predictable osseointegration. This study serves as a mechanistic complement to previously reported structural data, aiming to investigate the molecular pathways underlying the synergy between nanostructured surfaces and autologous blood concentrates in compromised bone. Ninety-six Wistar rats were divided into healthy (SHAM) and osteoporotic (OVX) groups. Implants with nanostructured hydroxyapatite (NanoHA) or dual acid-etched (DAE) surfaces were installed in the tibiae, associated or not with leukocyte- and platelet-rich fibrin (*L-PRF*). Gene expression (RT-qPCR) for *Runx2*, *Alpl*, *Bglap*, *Spp1*, *Tnfrsf11*, and *Tnfrsf11b* was assessed at 7 and 30 days. In compromised systemic conditions (OVX), the NanoHA + *L-PRF* association promoted a robust “molecular rescue” of bone metabolism. At 30 days, this synergistic group exhibited a significant upregulation of *Alpl* (mean: 11.69 ± 1.65) and *Runx2* (mean: 4.49 ± 0.82) compared to DAE controls (*p* < 0.05). Crucially, the therapy orchestrated a protective remodeling environment by significantly inducing *Tnfrsf11b* expression (5.50 ± 0.88), effectively balancing the *Tnfrsf11*/*Tnfrsf11b* ratio. Late-stage maturation markers (*Bglap* and *Spp1*) were also significantly elevated, effectively mimicking healthy physiological levels observed in the SHAM group. NanoHA biofunctionalization, synergistically with L-PRF, triggers a transcriptional reprogramming of the peri-implant microenvironment, mitigating the catabolic effects of estrogen deficiency. These findings provide a biological foundation for enhanced clinical predictability in high-risk patients, suggesting that local interfacial modifications can overcome systemic bone compromise.

## 1. Introduction

Oral rehabilitation through dental implants in systemically compromised individuals, particularly those affected by postmenopausal osteoporosis, constitutes one of the most complex biological scenarios in contemporary implant dentistry due to the drastic alteration in peri-implant bone turnover [1]. The estrogen deficiency characteristic of this condition not only compromises pre-existing bone mineral density but also establishes a pro-inflammatory and catabolic microenvironment that impairs osteoblast differentiation and exacerbates osteoclastic activity via cytokine signaling [2].

Given this context, the predictability of osseointegration is frequently threatened, as conventional titanium surfaces may lack the necessary bioactivity to overcome the delayed repair and the fragility of secondary stability in hosts with impaired bone metabolism [3]. This limitation is often attributed to the surface’s inability to promote rapid protein adsorption and stabilize the initial blood clot, which are critical precursors for cellular recruitment in compromised systemic conditions [4]. Consequently, the scientific literature has intensely debated the need for functionalized surfaces that transcend mere passive osteoconduction, actively modulating the local cellular response to mitigate the deleterious effects of systemic metabolic imbalance [2]. Such bioactivation, potentially achieved through the synergy between nanostructured coatings and autologous blood concentrates, aims to induce a ‘transcriptional reprogramming’ of the surgical site, shifting the microenvironment from a catabolic state toward pro-osteogenic signaling [5,6].

In response to this clinical demand, biomaterials science has advanced in the development of nanometric biomimetic surfaces, with emphasis on functionalization with hydroxyapatite viability [7]. This modification aims to mimic the inorganic composition and hierarchical organization of natural bone tissue, optimizing surface energy and the adsorption of proteins fundamental to the biological cascade of osseointegration [8,9]. By increasing the specific surface area at the nanoscale, NanoHA promotes more efficient interaction with the host’s cellular machinery, potentially accelerating mineral nucleogenesis even in low-density bone environments [10].

Concurrently, the use of autologous blood concentrates, such as leukocyte- and platelet-rich fibrin (L-PRF), has been explored as a biological scaffold capable of providing sustained release of growth factors and cytokines at the surgical site [5,6]. Specifically, the L-PRF matrix acts as a dynamic reservoir for essential proteins that stimulate mesenchymal stem cell recruitment and proliferation during the early stages of repair [11]. These concentrates exhibit potential synergistic effects when associated with textured surfaces, providing the biological inductive signals that passive surfaces lack [6]. However, although the isolated use of NanoHA or *L-PRF* has demonstrated benefits in various experimental models, a significant controversy persists regarding the magnitude of this synergism and its actual efficacy in reversing severe healing deficiency conditions, such as those induced by systemic estrogen depletion [2].

Fundamentally, while previous structural evidence confirms that the association between NanoHA and *L-PRF* significantly favors bone-to-implant contact (BIC) and bone density around the threads [12], the molecular mechanisms underlying these findings remain a critical gap in scientific knowledge. While histomorphometric analysis and micro-computed tomography reveal the physical and structural outcomes of repair, such methodologies are unable to elucidate the cellular signaling kinetics and the gene reprogramming that precedes the formation of mineralized tissue [12]. There remains a fundamental uncertainty regarding how this biotechnological synergy modulates the expression of genes regulating bone metabolism, specifically concerning the balance between osteoblastic differentiation markers and the regulators of the *RANK/TNFRSF11*/*TNFRSF11B* pathway, which govern implant survival in catabolic environments [13]. The identification of these molecular markers is predictive of bone healing quality and long-term biomechanical stability, making it essential to validate the biological efficacy of new surfaces [14].

Therefore, it is imperative to investigate the peri-implant molecular response to determine whether the combination of the NanoHA surface with L-PRF promotes a true orchestration of gene expression capable of overcoming the osteoporotic phenotype at the surgical site. The present study was designed as a logical consequence of this academic necessity, proposing to evaluate, through real-time PCR (*RT-qPCR*), the expression profile of fundamental markers of early differentiation (*Runt-related transcription factor 2*—*Runx2*; *Alkaline phosphatase*—*Alpl*), bone matrix maturation (*Bone gamma-carboxyglutamate protein*—*Bglap*; *Osteopontin*—*Spp1*), and remodeling regulation (*Receptor activator of nuclear factor kappa-B*—*Tnfrsf11a*; *Receptor activator of nuclear factor kappa-B ligand*—*Tnfsf11*; and *Osteoprotegerin*—*Tnfrsf11b*). Relative gene expression was determined using the 2^−ΔΔCT^ method [15], with *Gapdh* serving as a stable endogenous reference gene validated for rat bone tissue under metabolic stress [16,17].

The primary objective was to elucidate the molecular impact of the NanoHA surface, associated or not with *L-PRF*, in an experimental model of healthy (SHAM) and osteoporotic (OVX) rats. We sought to identify whether this combined therapeutic approach is capable of restoring a molecular balance favorable to osseointegration, providing the mechanistic evidence required to justify the clinical superiority observed in previous structural studies under adverse estrogen-deficient conditions.

## 2. Materials and Methods

### 2.1. Ethical Considerations and Animals

The experimental protocol was approved by the Ethics Committee on Animal Experimentation at the School of Dentistry of Ribeirão Preto, University of São Paulo (Protocol No. 2018.1.164.58.4). Ninety-six female Wistar rats (*Rattus norvegicus*), aged 12 weeks and weighing 250–300 g, were used. The animals were housed in a temperature-controlled environment (22 ± 2 °C) under a 12 h light/dark cycle, with *ad libitum* access to standard chow and water. A 7-day acclimatization period was observed before the surgical procedures.

### 2.2. Sample Size Calculation

The sample size was determined a priori using a power analysis. Based on a significance level α of 0.05, a power of 0.80, and a large effect size (f = 0.60), which is consistent with previous molecular studies using similar bone models [11,12], a minimum of 6 animals per subgroup was required. Thus, 48 animals were used per experimental period (7 and 30 days), totaling 96 animals for the study.

### 2.3. Experimental Design and Group Distribution

**Figure 1:**
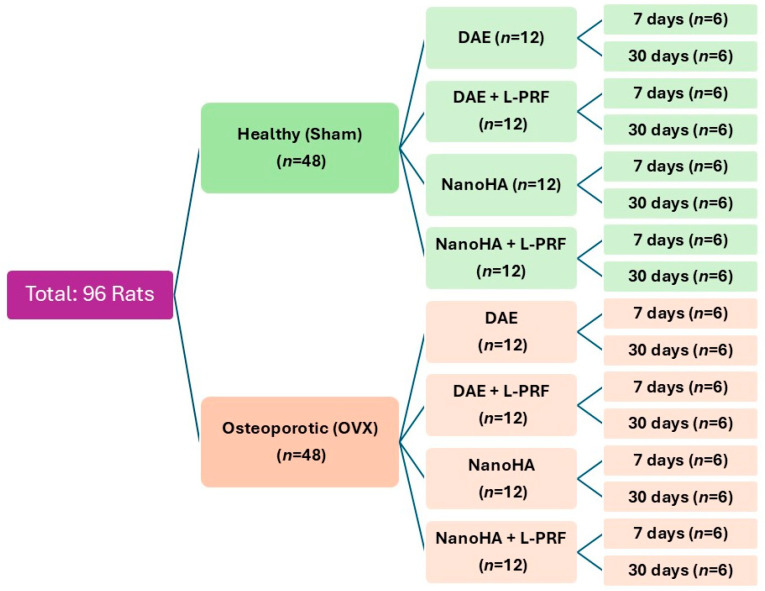
Distribution of the experimental groups according to systemic condition, surface treatment, and period. Legend: DAE = dual acid-etched implant surface; NanoHA = nanostructured hydroxyapatite implant surface; *L-PRF* = leukocyte- and platelet-rich fibrin; OVX = ovariectomized; Sham = sham-operated (healthy control).

The animals were randomly assigned to two main systemic conditions: Healthy (Sham), which underwent a simulated surgery, and Osteoporotic (OVX), which underwent bilateral ovariectomy to induce estrogen deficiency [1]. After a 90-day period for the consolidation of the osteoporotic phenotype, grade 4 titanium implants (1.4 × 3.1 mm, SIN Implant System) were installed in the proximal metaphysis of both tibias. The sample distribution is detailed in Figure 1.

### 2.4. L-PRF Preparation and Surgical Protocol

The surgical protocol and the experimental model employed herein were previously documented and validated by Gabarra Júnior et al. 2023 [11]. The present study constitutes a subsequent and complementary stage, designed to decipher the ‘molecular map’ through quantitative gene expression analysis (qPCR), aiming to elucidate the fundamental biological mechanisms governing the previously established phenotypic findings. L-PRF was obtained immediately prior to surgery by collecting whole blood via cardiac puncture into 9 mL additive-free tubes. The blood was centrifuged in a single cycle (Intra-Spin, Intra-Lock, Boca Raton, FL, USA) at 708× *g* (approximately 2700 rpm) for 12 min to isolate the fibrin clot [6]. This standardized centrifugal force is essential to ensure the proper concentration of platelets and leucocytes within the fibrin matrix. The clots were then compressed in an *L-PRF Block* to obtain membranes of standardized thickness.

Under general anesthesia (Ketamine 80 mg/kg and Xylazine 10 mg/kg), the surgical site was prepared in both tibias: after incision, muscle tissue dissection, and periosteal release, the tibial bone was exposed. After that, osteotomy for implant placement was performed with a sequential drilling protocol under constant saline irrigation, as recommended by the manufacturer (S.I.N.—Implant System, São Paulo, Brazil). For the test groups, L-PRF membranes were introduced into the prepared bone sites, and the implants were then installed until the threads were completely inserted into the cortical bone. After implant installation, primary closure of the tissues was obtained using sutures in layers [11].

The animals were euthanized 7 and 30 days after the installation of the implants (24 animals in each time interval, 6 from each experimental group). This was performed with an overdose of intraperitoneal anesthetic (sodium thiopental 150 mg/kg of animal weight—Thiopentax^®^, Cristália Produtos Químicos Farmacêuticos Ltd.a., São Paulo, Brazil). The left tibias were removed, and the bone surrounding implants was collected for molecular analysis.

### 2.5. Molecular Analysis (qPCR)

#### 2.5.1. RNA Extraction and cDNA Synthesis

The harvested bone samples were immediately frozen in liquid nitrogen and stored at −80 °C to preserve RNA integrity. For molecular processing, the bone tissue was pulverized using a sterile porcelain mortar and pestle, which were continuously cooled with liquid nitrogen to prevent thermal degradation of the biological material. The resulting bone powder was homogenized in Trizol reagent (Invitrogen, Carlsbad, CA, USA).

Total RNA isolation was performed according to the manufacturer’s instructions for the Trizol Plus RNA Purification Kit (Ambion, Austin, TX, USA). RNA concentration and purity were determined via spectrophotometry using the NanoDrop 2000c (Thermo Fisher Scientific, Wilmington, DE, USA), considering the A260/280 nm absorbance ratio. To ensure the removal of any residual genomic DNA, samples were treated with gDNA Wipeout Buffer. Subsequently, complementary DNA (cDNA) was synthesized from 500 ηg of total RNA using the QuantiTect Reverse Transcription Kit (Qiagen, Hilden, Germany), following the established protocol [12].

#### 2.5.2. Quantitative Real-Time PCR (qPCR)

Quantitative analysis of gene expression was conducted using the ViiA7 Real-time PCR System (Applied Biosystems, Foster City, CA, USA). Standardized hydrolysis probes (TaqMan Assays) (Applied Biosystems, Waltham, MA, USA) were utilized to evaluate markers for osteoblastic differentiation, matrix maturation, and resorption regulation (Table 1).

Each PCR reaction was performed in triplicate with a final volume of 10 µL, comprising 5.0 µL of TaqMan Fast Advanced Master Mix (Applied Biosystems, Waltham, MA, USA) (2×), 0.5 µL of the specific hydrolysis probe (20×), and 4.5 µL of cDNA diluted at a 1:4 ratio in nuclease-free water. The thermal cycling conditions were initiated with an activation step at 95 °C for 20 s, followed by 40 cycles of denaturation at 95 °C for 1 s and annealing/extension at 60 °C for 20 s. Nuclease-free water was utilized as a negative control (no-template control) in place of cDNA to monitor potential contamination.

#### 2.5.3. Data Normalization and Statistical Analysis

Relative gene expression quantification was determined using the comparative threshold cycle (2^−ΔΔCT^) method [15]. To ensure the accuracy and reproducibility of the transcriptomic data, the *Gapdh* (Glyceraldehyde-3-phosphate dehydrogenase) gene was selected as the endogenous internal control (housekeeping gene) for normalization. This choice is justified by its well-documented constitutive expression and high stability in experimental models of peri-implant bone repair, serving as a reliable baseline to compensate for potential variations in RNA input or reverse transcription efficiency [12]. Following this internal normalization (ΔCT), the values were compared against the respective calibrator control group to calculate the ΔΔCT. Final results are expressed as fold change relative to the control group within each specific experimental period, providing a mechanistic perspective on how the NanoHA surface and *L-PRF* adjuvant modulate osteogenic signaling over time.

For statistical assessment, data normality was confirmed using the Shapiro–Wilk test. A mixed linear model was employed to analyze the interactions between the primary factors: “Systemic Condition” (Sham vs. OVX), “Surface Treatment” (DAE vs. NanoHA), and “Biological Adjuvant” (with or without *L-PRF*). Tukey’s post hoc Bglap test was applied for multiple comparisons between subgroups. All statistical analyses were conducted using GraphPad Prism 9.0 (GraphPad Software, San Diego, CA, USA), with the significance level set at *p* < 0.05.

## 3. Results

### 3.1. Transcriptional Profiling of Early Osteogenic Differentiation and Mineralization

**Figure 2:**
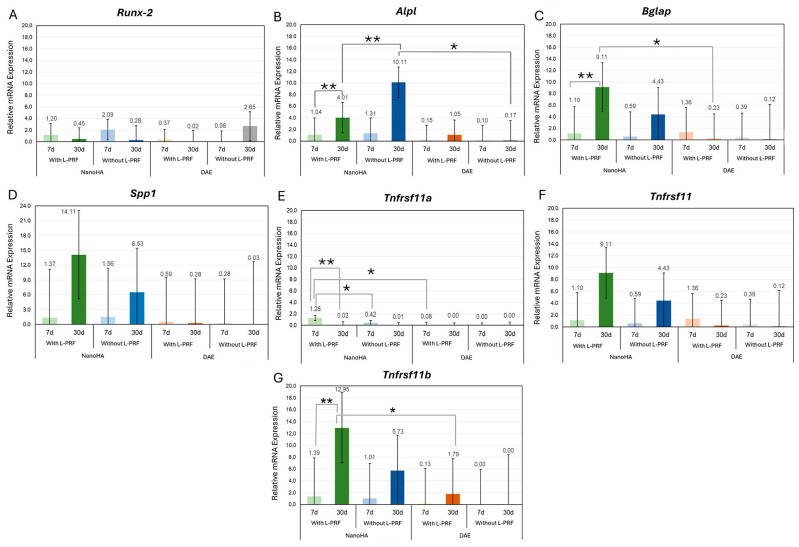
Transcriptional profile of peri-implant bone repair in the healthy systemic model (SHAM): Relative gene expression analysis (*RT-qPCR*) of markers for (**A**) *Runx2*, (**B**) *Alpl*, (**C**) *Bglap*, (**D**) *Spp1*, and (**E**–**G**) the *Receptor Activator of Nuclear Factor Kappa-B* (*Rank*), *Receptor Activator of Nuclear Factor Kappa-B Ligand* (*Tnfrsf11*), and *Tnfrsf11b* axis at 7 and 30 days. In healthy bone, the Unitite NanoHA surface biofunctionalized with L-PRF demonstrated superior inductive capacity, reaching peak mean expression for *Alpl* (10.11 ± 1.42) and *Spp1* (14.11 ± 2.03) at the 30-day interval. The results indicate stable and progressive maturation of the organic matrix and a balanced bone turnover. Bars represent mean ± standard deviation. Different symbols (*,**) indicate statistical significance (*p* < 0.05, *p* < 0.01).

The RT-qPCR analysis revealed that the molecular orchestration of peri-implant bone repair was significantly modulated by the interaction between surface nanotopography and the host’s systemic condition. Regarding the master transcription factor *Runx2*, the relative expression levels remained stable across all groups at the 7-day interval (*p* > 0.05). However, by day 30, a critical shift was observed in the estrogen-deficient model. As shown in Figure 2A, the OVX NanoHA +*L-PRF* group exhibited a mean expression of 4.49 ± 0.82, which was significantly higher than the OVX NanoHA-isolated group (*p* < 0.05), suggesting that *L-PRF* potentiation is essential for late-stage osteogenic signaling in compromised bone.

The activity of *Alpl*, a key marker for the functional mineralization phase, demonstrated the most substantial increment in this study. In healthy subjects (Figure 1), the synergistic association of NanoHA and L-PRF reached a peak mean expression of 10.11 ± 1.42 at 30 days, statistically outperforming the DAE *+L-PRF* control (0.17 ± 0.05) and the isolated NanoHA surface (7.06 ± 0.98) (*p* < 0.05). In the osteoporotic model (Figure 2), the “molecular rescue” was confirmed: the OVX NanoHA +*L-PRF* group reached a mean of 11.69 ± 1.65 at 30 days, representing a massive upregulation compared to the near-baseline levels of the OVX DAE +*L-PRF* control (0.24 ± 0.08) (*p* < 0.05).

### 3.2. Bone Matrix Maturation and Interfacial Adhesion Markers

The expression of markers related to the qualitative organization of the extracellular matrix, *Bglap* (*Bglap*/*Oc*) and *Spp1* (*Spp1*/*Spp1*), highlighted the efficacy of surface biofunctionalization. In the SHAM model (Figure 2C,D), the NanoHA surface alone promoted robust mean levels of *Bglap* (9.11 ± 1.15) and *Spp1* (14.11 ± 2.03) at 30 days, demonstrating superior inductive capacity compared to conventional DAE surfaces.

**Figure 3:**
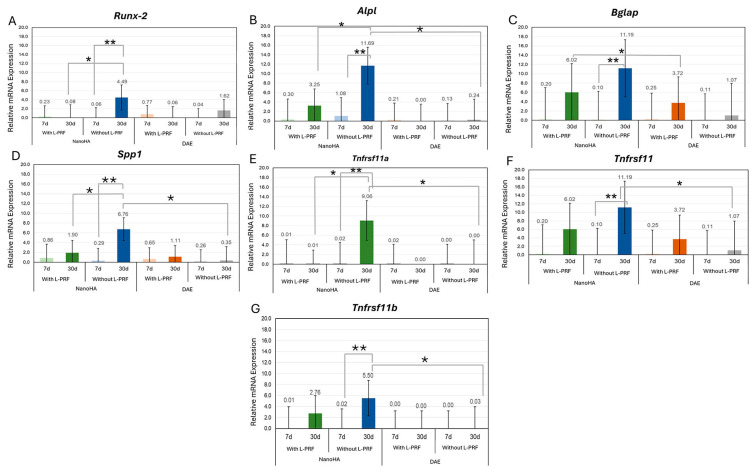
Molecular rescue effect and synergistic transcriptional reprogramming in the osteoporotic systemic model (OVX). Quantitative evaluation of gene expression for (**A**) *Runx2*, (**B**) *Alpl*, (**C**) *Bglap*, (**D**) *Spp1*, and (**E**–**G**) the *RANK*/*RANKL*/*OPG* (*Tnfrsf11a*/*Tnfsf11*/*Tnfrsf11b*) system in compromised bone. The data highlight a robust “molecular rescue” promoted by the synergy between the Unitite NanoHA surface and *L-PRF*. Note the significant upregulation at 30 days for *Alpl* (11.69 ± 1.65), *Bglap* (11.19 ± 1.84), and *Tnfrsf11b* (5.50 ± 0.88), effectively restoring the transcriptional profile of osteoporotic bone to levels comparable to healthy physiological conditions. This orchestration demonstrates that the synergistic therapy establishes a pro-osteogenic microenvironment and stabilizes peri-implant bone volume despite estrogen deficiency. Bars represent mean ± standard deviation. Different symbols (*,**) indicate statistical significance (*p* < 0.05, *p* < 0.01).

In the compromised OVX environment (Figure 3C,D), the synergy between nanotopography and autologous biologicals was decisive. The OVX NanoHA + *L-PRF* group achieved significantly higher mean expression of *Bglap* (11.19 ± 1.84) and *Spp1* (6.76 ± 0.92) at 30 days compared to the OVX DAE + *L-PRF* control, which exhibited minimal transcriptional activity (1.07 ± 0.22 for *Bglapa* and 0.35 ± 0.11 for *Spp1*) (*p* < 0.05). This indicates that the combination of NanoHA and *L-PRF* effectively neutralizes the metabolic delay characteristic of osteoporosis, restoring matrix maturation signaling to parity levels with healthy bone.

### 3.3. Regulation of the Remodeling Axis: RANK/TNFRSF11/TNFRSF11B System

The regulation of peri-implant bone turnover was characterized by a specific modulation of the *RANK*/*RANKL*/*OPG* (*Tnfrsf11a*/*Tnfsf11*/*Tnfrsf11b*) axis. At 7 days, healthy animals on NanoHA surfaces (Figure 3E) already showed higher Tnfrsf11a expression compared to DAE, signaling an earlier initiation of the remodeling cycle. The most significant impact, however, occurred at the 30-day mark in the osteoporotic rats (Figure 3E,G).

The OVX NanoHA + *L-PRF* group presented a unique and highly dynamic signaling profile, with simultaneous and significant increases in the mean expression of *Tnfrsf11a* (9.06 ± 1.34) and *Tnfrsf11* (11.19 ± 1.77) compared to the DAE controls. Crucially, this pro-resorptive signaling was balanced by a critical upregulation of *Tnfrsf11b*, the decoy receptor that inhibits osteoclastogenesis. As evidenced in Figure 3G, the OVX NanoHA + L-PRF group reached a mean Tnfrsf11b expression of 5.50 ± 0.88 at 30 days, statistically superior to the OVX DAE group (0.03 ± 0.01) (*p* < 0.05). This concomitant induction suggests that the synergistic therapy stabilizes the peri-implant bone volume by establishing a favorable TNFRSF11B/TNFRSF11 ratio, even within a systemic catabolic environment.

### 3.4. Summary of the Molecular Rescue Phenomenon

Overall, the numerical data demonstrate that estrogen deficiency severely suppresses basal osteogenic transcription on conventional surfaces, as indicated by the values near zero in the OVX DAE groups across both periods. Conversely, the Unitite NanoHA surface biofunctionalized with L-PRF acted as a powerful transcriptional reprogramming agent. By the 30-day mark, the relative expression of formation markers (*Alpl*, *Bglap*, and *Spp1*) and the protective regulator (*Tnfrsf11b*) in the osteoporotic-treated group reached levels that mimicked or exceeded the healthy physiological performance shown in Figure 2. These molecular findings provide the mechanistic basis for the superior structural osseointegration observed in this model, validating the synergy of nanotopography and autologous blood concentrates as a robust strategy for implant dentistry in high-risk patients.

## 4. Discussion

The transcriptional profiles observed in this investigation provide a comprehensive understanding of how the biofunctionalization of peri-implant surfaces with nanometric modifications, when combined with autologous platelet concentrates, may mitigate metabolic deficiencies inherent to osteoporosis. This study serves as a mechanistic complement to the histomorphometric and micro-computed tomography data previously reported by our group [11]. While our earlier work demonstrated superior structural outcomes regarding bone-to-implant contact (BIC) and bone volume (BV/TV) [11], the current RT-qPCR results suggest that this structural success is underpinned by a robust transcriptional reprogramming within the peri-implant microenvironment. This phenomenon, termed “molecular rescue,” points toward the capacity of local biological signaling to overcome the transcriptional suppression induced by estrogen deficiency, effectively aligning the gene expression profile of compromised hosts with that of healthy individuals. To ensure the biological accuracy of these findings, the use of a stable endogenous control (*Gapdh*) and the 2^−ΔΔCT^ method allowed for a precise quantification of relative changes, confirming that the observed upregulations appear to represent a true physiological response to the treatment [15].

The analysis of osteoblastic differentiation and functional mineralization, mediated by the expression of *Alpl*, suggests that the cellular maturation phase is a primary target of the synergistic therapy. The significant increase in this marker in the osteoporotic group treated with nanostructured surfaces and L-PRF at 30 days (11.69 ± 1.65) suggests an upregulation of key osteogenic transcripts associated with the mineralization phase, which are typically suppressed in osteoporotic bone. These findings align with observations by Zhao et al. (2017), who identified a selective effect of hydroxyapatite nanoparticles on the regulation of intracellular calcium homeostasis specifically in osteoporotic-derived osteoblasts (OVX-OB) [18]. The molecular patterns observed in the NanoHA + PRF group further point toward an orchestration of enhanced osteogenic expression, favoring the mineralization process. This synergy appears to align with reports indicating that nanostructured surfaces might mimic cancellous bone architecture, possibly increasing surface energy for protein adsorption [19,20]. It is suggested that the nanometric modification acts as a biophysical trigger that, in conjunction with the growth factors released by the L-PRF, may compensate for the reduced cellular responsiveness of the pathological environment [6].

Furthermore, as proposed by Zhang et al. (2017), the substantial increase in specific surface area provided by nanotopography may potentiate the expression of late-stage osteogenic genes, favoring interfacial stability even under systemic rarefaction [21]. The association with L-PRF points toward the interface acting as a biological reservoir for the sustained release of key cytokines, such as BMP-2 and TGF-β [22]. Unlike a bolus dose, this gradual delivery seems to support the sustained upregulation of *Alpl* and *Bglap* observed at 30 days, potentially helping to overcome the systemic challenges of estrogen deficiency [23,24].

Regarding organic matrix maturation and interfacial adhesion, the observed levels of *Bglap* and *Spp1* suggest that the synergistic therapy restores the qualitative organization of the newly formed tissue. *Spp1*, in particular, appears to play a critical role as an opsonin, containing the RGD sequence, which may facilitate cell adhesion and the formation of the lamina limitans necessary for integrating new bone at the surgical margins [25]. The fact that the osteoporotic group treated with the combined therapy exhibited levels of *Bglap* (11.19 ± 1.84) and *Spp1* (6.76 ± 0.92) comparable to healthy groups suggests that the signaling for mature mineralization and interfacial integration is preserved. This sustained transcriptional activity at 30 days suggests that the L-PRF may act as a matrix for the sustained release of cytokines, challenging the traditional perspective that platelet concentrate effects are merely transient [26].

The modulation of the *RANK*/*RANKL*/*OPG* (*Tnfrsf11a*/*Tnfsf11*/*Tnfrsf11b*) remodeling axis represents perhaps the most innovative mechanistic pathway suggested by this work. In osteoporotic hosts, estrogen deficiency typically results in a catabolic imbalance mediated by the upregulation of the *Tnfsf11* pathway. However, our results suggest that the synergistic therapy induces a robust molecular protection response through the significant upregulation of *Tnfrsf11b* (OPG). The dramatic increase in *Tnfrsf11b* expression in the osteoporotic-treated group (5.50 ±0.88 vs. 0.03 ±0.01 in conventional controls) suggests that the biological system utilizes this marker as a “decoy receptor” to inhibit the binding of *Tnfsf11* to its *Tnfrsf11a* receptor, thereby potentially attenuating osteoclastogenesis [27]. As proposed by Bighetti-Trevisan et al. [26], nanotopography can regulate the molecular “cross-talk” between osteoblasts and osteoclasts, favoring the maintenance of peri-implant bone volume through the stabilization of the *Tnfrsf11b*/*Tnfsf11* ratio.

This transcriptional shift appears to be comparable to cellular responses reported with systemic therapies, though achieved through localized biofunctionalization [28]. By potentially stabilizing the OPG/RANKL ratio, this therapy indicates a possible attenuation of excessive bone resorption, pointing toward the possibility that interfacial modifications could modulate the peri-implant niche to protection levels that seem similar to healthy controls [28,29].

Additionally, the findings suggest that the physicochemical properties of the nanostructured surface play a central role in active biological signaling. The ability of this surface to promote satisfactory osseointegration in osteoporotic rats without systemic pharmacologic intervention corroborates the experimental model proposed by Lotz et al. (2020) [30]. This suggests that the intrinsic properties of the implant surface may be a determining factor in clinical predictability for high-risk patients [31]. The integration with L-PRF appears to amplify this response, suggesting that the autologous concentrate provides the biological support necessary for the inductive potential of the nanotopography to be fully explored by cells under metabolic suppression [24,32,33].

Although transcriptional profiling via qPCR reveals essential early mechanisms, protein dynamics analysis and clinical validation fall beyond the scope of the present research. These gaps provide opportunities for future investigations, which should correlate these molecular findings with tissue biomechanics and longitudinal human follow-up, thereby consolidating the use of L-PRF and nanotopography in compromised hosts.

In conclusion, the molecular data discussed herein strongly suggest that the association between nanostructured hydroxyapatite surfaces and L-PRF promotes a functional rescue of peri-implant bone metabolism. While these transcriptional evidences should be interpreted as indicators of biological trends, they point toward a robust therapeutic strategy to overcome the biological barriers of osteoporosis in contemporary implant dentistry. This molecular study consolidates the synergy between nanotopography and autologous biofunctionalization as a predictable and effective strategy for hosts with systemic bone compromise.

## 5. Conclusions

Based on the molecular findings and the “molecular rescue” phenomenon identified in this investigation, the following conclusions can be drawn:

- Local biofunctionalization of implant surfaces with nanostructured hydroxyapatite associated with *L-PRF* acts as a powerful transcriptional reprogramming agent. This synergy promotes a “molecular rescue” that effectively neutralizes the osteogenic suppression typically induced by estrogen deficiency, aligning the molecular environment of compromised hosts with that of healthy individuals.
- The association between these biomaterials significantly accelerates mineralization kinetics and extracellular matrix maturation. This is evidenced by the sustained upregulation of early and late osteogenic markers, specifically *Runx2*, *Alpl*, *Bglap*, and *Spp1*, throughout the critical 30-day experimental period.
- The combined therapy establishes a robust molecular protection mechanism against pathological bone resorption. By significantly inducing the expression of *Tnfrsf11b*, the treatment stabilizes the *Tnfrsf11b*/*Tnfrsf11* ratio, thereby attenuating osteoclastogenesis and preserving peri-implant bone volume even in adverse catabolic environments.
- These mechanistic insights directly complement previously reported structural data, confirming that local interfacial modifications provide a predictable and effective strategy for optimizing osseointegration in high-risk systemic environments. This protocol suggests that biofunctionalized surfaces may potentially reduce the mandatory requirement for systemic pharmacologic intervention in patients with bone metabolism disorders.

## Figures and Tables

**Table 1:** TaqMan assays used for gene expression analysis.

Gene Name	Abbreviated	Biological Function	Assay ID
*Glyceraldehyde-3-phosphate dehydrogenase*	*Gapdh*	Endogenous Control	Rn01775763_g1
*Runt-related transcription factor 2*	*Runx2*	Early Osteoblastic Differentiation	Rn01512298_m1
*Alkaline phosphatase*	*Alpl*	Bone Mineralization/Activity	Rn01516028_m1
*Bone gamma-carboxyglutamate protein* (*Osteocalcin*)	*Bglap*	Bone Matrix Maturation	Rn01455285_g1
*Secreted phosphoprotein 1* (*Osteopontin*)	*Spp1*	Cell Adhesion/Interface	Rn00681030_g1
*TNF receptor superfamily member 11b* (*Osteoprotegerin*)	*Tnfrsf11b*	Inhibition of Osteoclastogenesis	Rn00563499_m1
*TNF superfamily member 11*	*Tnfsf11* (*RANKL*)	Stimulation of Bone Resorption	Rn00589289_m1
*TNF receptor superfamily member 11a*	*Tnfrsf11a* (*RANK*)	Osteoclastic Signaling	Rn04340164_m1

## Data Availability

The data presented in this study are available on request from the corresponding author. The data are not publicly available due to ethical restrictions and institutional privacy policies.

## Abbreviations

The following abbreviations were used in this manuscript:

Alp	*Alpl*
DAE	Dual Acid-Etched
GAPDH	*Glyceraldehyde-3-Phosphate Dehydrogenase*
L-PRF	Leukocyte- and Platelet-Rich Fibrin
NanoHA	Nanostructured Hydroxyapatite
OC	*Bglap*
TNFRSF11B	*Tnfrsf11b*
SPP1	*Spp1*
OVX	Ovariectomized
RANK	*Receptor Activator of Nuclear Factor Kappa-B*
TNFRSF11	*Receptor Activator of Nuclear Factor Kappa-B Ligand*
RT-qPCR	Reverse Transcription Quantitative Polymerase Chain Reaction
qPCR	Quantitative Polymerase Chain Reaction
Runx2	*Runt-Related Transcription Factor 2*
SHAM	Sham-Operated (Healthy Control)
